# Plasma N-Acetylaspartate Is Related to Age, Obesity, and Glucose Metabolism: Effects of Antidiabetic Treatment and Bariatric Surgery

**DOI:** 10.3389/fendo.2020.00216

**Published:** 2020-04-17

**Authors:** Giuseppe Daniele, Beatrice Campi, Alessandro Saba, Simone Codini, Annamaria Ciccarone, Laura Giusti, Stefano Del Prato, Russel L. Esterline, Ele Ferrannini

**Affiliations:** ^1^Department of Clinical and Experimental Medicine, University of Pisa, Pisa, Italy; ^2^C.N.R. Institute of Clinical Physiology, Pisa, Italy; ^3^Department of Surgical, Medical and Molecular Pathology and Critical Care Medicine, University of Pisa, Pisa, Italy; ^4^Laboratory of Clinical Pathology, St. Chiara University Hospital, Pisa, Italy; ^5^AstraZeneca Pharmaceutical, Gaithersburg, MD, United States

**Keywords:** N-acetylaspartate, neuronal function, type 2 diabetes, obesity, dapagliflozin, exenatide, bariatric surgery, glucose metabolism

## Abstract

**Background:** N-acetylaspartate (NAA) is synthesized only by neurons and is involved in neuronal metabolism and axonal myelination. NAA is the strongest signal on brain magnetic resonance spectroscopy, and its concentration have been associated with cognitive dysfunction in neurodegenerative diseases, obesity, and type 2 diabetes (T2D).

**Materials and Methods:** We explored the impact of obesity and T2D on circulating NAA as well as the impact of bariatric surgery and antidiabetic treatments. We developed an LC-MS method for the accurate measurements of fasting plasma NAA levels in 505 subjects (156 subjects with normal glucose tolerance, 24 subjects with impaired glucose tolerance, and 325 patients with T2D) to examine the associations of NAA with obesity and dysglycemia. To validate cross-sectional findings, plasma NAA was measured 6 months after Roux-en-Y Gastric Bypass (RYGB) in 55 morbidly obese subjects, and after 1 year of antidiabetic treatment (with dapagliflozin, exenatide, or dapagliflozin plus exenatide) in 192 T2D patients.

**Results:** In the whole population, NAA was associated with age (*r* = 0.31, *p* <0.0001) and BMI (*r* = −0.20, *p* <0.0001). Independently of age and BMI, NAA was reciprocally related to HbA1c and fasting plasma glucose (partial *r* = −0.13, both *p* = 0.01). Surgically-induced weight loss raised NAA (by 18 nmol/L on average, *p* <0.02). Glucose lowering treatment increased NAA in proportion to the drop in HbA1c (*r* = 0.31, *p* <0.0001) regardless of the agent used.

**Conclusions:** Circulating NAA concentrations are modulated by age, obesity, and glycemic control. Whether they may mark for the corresponding metabolic effects on brain function remains to be established by joint measurements of spectroscopic signal and cognitive function.

## Introduction

Type 2 diabetes (T2D) and obesity increase the risk of serious complications in multiple organs, including the brain (1). Obesity and T2D are associated with a higher prevalence of cognitive dysfunction, dementia, and neurodegenerative diseases, including Alzheimer and Parkinson diseases (2–5). Microvascular disease induced by hyperglycemia is responsible for disruption of the blood-brain barrier (BBB) through pericyte depletion, possibly caused by excess superoxide produced during enhanced mitochondrial respiration (6, 7). Loss of the BBB ability to protect the brain from circulating substances results in neurotoxicity and neuronal cell death (8). Neuroimaging has documented several structural and functional alterations in numerous brain regions of patients with T2D (9–11). Magnetic resonance spectroscopy (MRS) has provided details regarding metabolite changes in the brain that might be related to functional and structural alterations. Recent MRS-based analyses carried out in humans have suggested that N-acetylaspartate (NAA), the most concentrated metabolite in the brain (~10 mM) (12–14), is significantly reduced in several diseases including Alzheimer (15, 16), Huntington disease (17), bipolar disorder (18), schizophrenia (19), multiple sclerosis (20), and T2D (21–25).

NAA is reputed to be a marker of neuronal number and viability (26) but its role in brain metabolism and function in humans is far from being fully elucidated. NAA is synthesized in neuronal mitochondria and then transported by a sodium/dicarboxylate symporter (27) to aspartoacylase-containing oligodendrocytes (28) and astrocytes. NAA has two primary roles, as a facilitator of energy metabolism in neuronal mitochondria (29) and a source of acetate for fatty acid and steroid synthesis necessary for axonal myelination by oligodendrocytes (30, 31). Astrocytes, which represent the main cell type within the BBB structure, play a role in the removal of NAA from the extracellular space; a continuous NAA efflux from the brain to the circulation has been proposed (32). Therefore, plasma NAA levels might be used as a marker of central NAA alterations in neurodegenerative diseases and T2D-related brain damage. One difficulty is that the few studies measuring circulating NAA concentrations report widely different values even in healthy controls, ranging from a mean 0.11 mmol/L (33) to 0.44 μmol/L (34), very likely due to assay differences.

To the best of our knowledge, the impact of obesity and T2D on circulating NAA has not been previously explored. We therefore set forth to measure plasma NAA in non-diabetic obese and T2D subjects and the effect of treatment on plasma NAA.

## Materials and Methods

### Study Subjects

This was a data pooling project of different cohort studies. A total of 505 study subjects included patients with T2D or impaired glucose tolerance (IGT) and non-diabetic obese subjects. Two subgroups of participants were studied: (i) to assess the impact of major weight loss on circulating NAA levels, we included obese subjects undergoing Roux-en-Y Gastric Bypass (RYGB) between 2015 and 2018; (ii) to evaluate the effect of glycemic control, we included T2D patients from the DURATION-8 study (NCT02229396), which was a 52-week, multicenter, double-blind, randomized, active-controlled phase 3 trial. In DURATION-8, participants were randomly assigned to receive once-weekly exenatide (2 mg) by subcutaneous injection plus once-daily dapagliflozin (10 mg) (EQW+Dapa), exenatide with dapagliflozin-matched oral placebo (EQW), or dapagliflozin with exenatide-matched placebo injections (Dapa+Plb) (35). Lean healthy subjects with normal glucose tolerance (NGT) were included as control group. Exclusion criteria were: type 1 diabetes, psychiatric disorders, severe cognitive impairment, neurodegenerative diseases, epilepsy, depression treatment, traumatic brain injury over the preceding months, liver function enzymes higher more than two times the upper limit, heart failure (NYHA III-IV), GFR <60 ml/min/1.73 m^2^, diabetes treatment other than metformin, or sulphonylureas. The ethical committee approvals refers to specific ethical approval of each study included in the pooled analysis. All procedure performed were in accordance with the Helsinki Declaration of 1975 as revised 1983.

All plasma samples were obtained in the overnight fasted [10–14 h) state, and stored at −80°C for NAA analysis. Baseline and post-treatment samples were assayed in the same run to reduce within-subject variability.

### Reagents and Materials

NAA and internal standard *N*-Acetyl-L-aspartic acid-1,2,3,4-^13^C_4_ (IS, ^13^C_4_-NAA) as well as acetonitrile (LC-MS grade), ultra-pure water (LC-MS grade), formic acid (MS grade), and 3N hydrochloric acid in butan-1-ol were provided by Sigma-Aldrich (Saint Louis, MO, USA).

### Equipments and Analytical Method

An Agilent 1290 Infinity UHPLC system (Santa Clara, CA, USA), including binary pump, autosampler, and column oven, fitted with a Kinetex C18 (100Å, 2.6 μm, 50 ×2.10 mm ID) HPLC column (Phenomenex, Torrance, CA, USA), protected by a KrudKatcher Ultra HPLC in-line filter (0.5 μm depth ×0.004 in ID), was used for sample injection and chromatographic separation. This latter was carried out under the gradient conditions at a flow rate of 400 μL/min, by using H_2_O + 0.1% formic acid (FA) as an aqueous solvent and ACN + 20% MeOH + 0.1% FA as an organic solvent. The injection volume of samples was 5 μL.

Mass spectrometry runs were carried out by an AB Sciex API 4000 triple quadrupole mass spectrometer (Concord, ON, Canada), equipped with an electrospray (ESI) Turbo-V ion source. The MS method was based on positive ion mode selected reaction monitoring (SRM) and made use of parameters optimized in order to get the best possible sensitivity and selectivity. In particular, SRM acquisitions were based on transitions 288.2 → 143.9 Da (quantifier, Q) and 288.2 → 186.1 Da (qualifier, q) for NAA and 292 → 146.9 Da (Q), 292 → 189.1 Da (q) for ^13^C_4_-NAA.

Sample preparation was carried out as follows: plasma samples were thawed at room temperature, vortexed (15 min), and a 100 μL aliquot was added with 300 μL of acetonitrile, formic acid 1% (V%), and internal standard. The obtained suspensions were vortexed (15 min) and centrifuged (18,620 × g, 15 min). Then, 300 μL of supernatants were collected and dried under stream of N_2_ at 40°C. The samples were derivatized to butyl esters (Fischer esterification reaction) by adding 100 μL 3N 1-butanol/ HCl, vortexing (15 min), heating at 60°C for 40 min. The reaction products were dried under a gentle stream of nitrogen and the dry residues were reconstituted with 100 μL ACN/H_2_O (20/80; V/V), vortexed (15 min), and 5 μL of the obtained solutions were injected into the LC-MS-MS system.

The analytical method, which is not the main subject of this paper and that will be extensively described in a paper under preparation, was validated in compliance with EMA guidelines (36) for sample stability, linearity, lower limits of detection and quantitation (LLOD and LLOQ), matrix effect, recovery, selectivity, precision, and accuracy.

### Statistical Analysis

Data are given as mean ± SD. Because of their skewed distribution, plasma NAA concentrations were summarized as median [interquartile range, IQR], and logarithmically transformed for use in parametric statistical tests. Group values were compared by the Mann-Whitney *U*-test, paired values by the Wilkinson signed-rank test. Change by treatment were analyzed by MANOVA for repeated measures. Univariate and multivariate linear regressions were carried out by standard methods. Data analyses were performed using JMP® 7.0 (SAS Institute Inc., 2007); *p* ≤ 0.05 was considered statistically significant.

## Results

**Figure 1:**
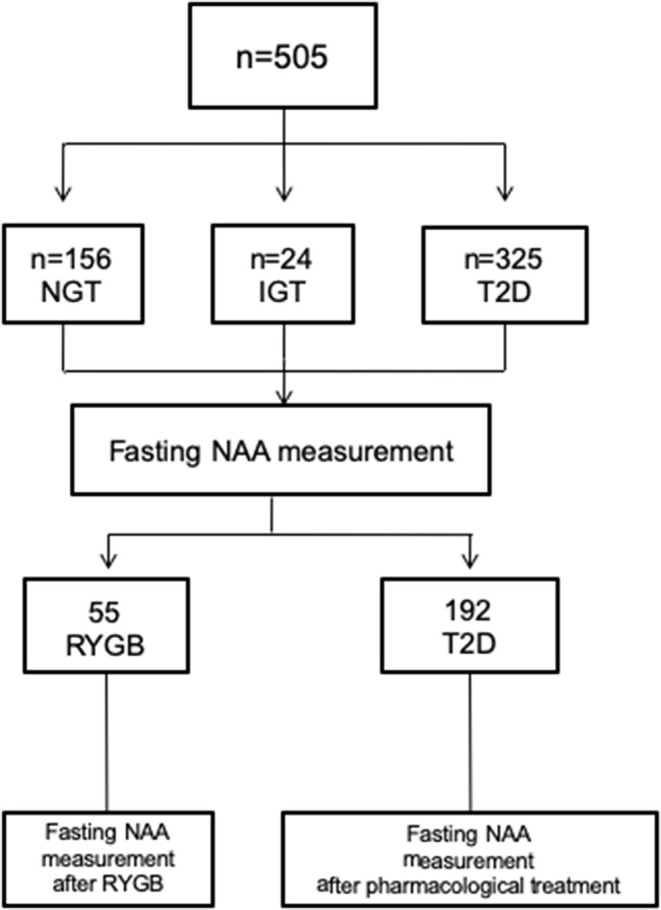
Participants flow chart. NGT, normal glucose tolerance; IGT, Impaired glucose regulation; T2D; type 2 diabetes.

**Figure 2:**
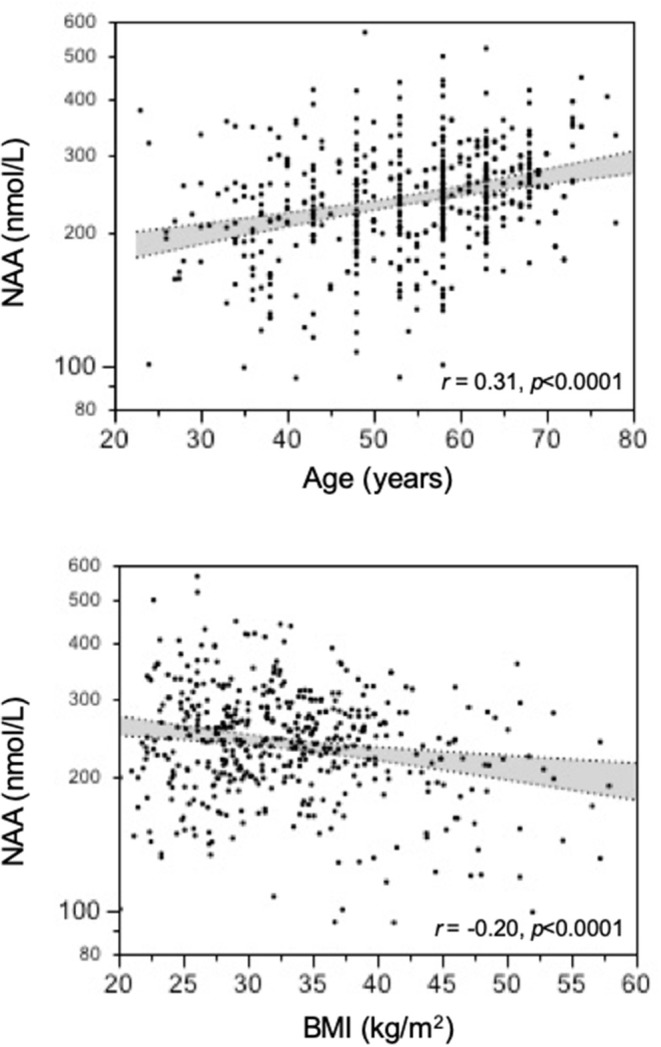
Association of plasma N-acetylaspartate (NAA) concentrations with age and BMI.

**Figure 3:**
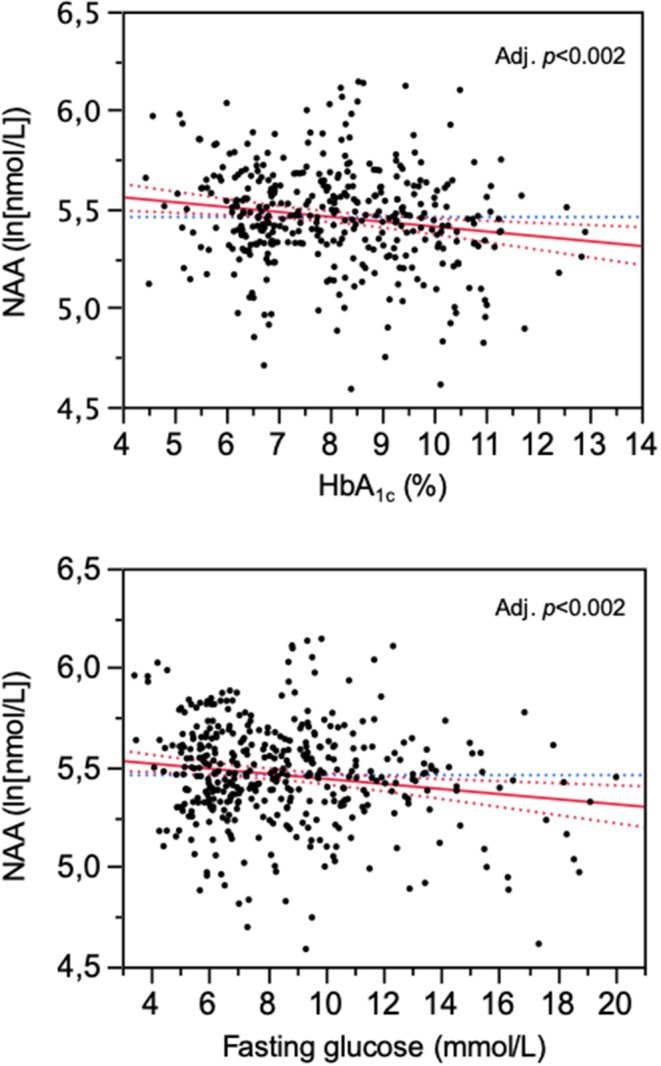
Association of plasma N-acetylaspartate (NAA) concentrations with HbA_1c_ and fasting glucose levels. The plots are for logNAA residuals after controlling for gender, age, and BMI.

In the study population (259 women, 246 men), 156 subjects had normal glucose tolerance (NGT), 325 had T2D, and 24 had IGT; age ranged from 23 to 78 years, and BMI ranged from 20.1 to 57.9 kg/m^2^ (Figure 1). In the whole data set, fasting plasma NAA concentrations ranged from 94 to 567 nmol/L, with a significantly (*p* <0.0001) skewed distribution. Compared to NGT, T2D/IGT subjects were older (*p* <0.0001) and more often men (*p* = 0.002) (Table 1). Plasma NAA concentrations were higher in men than women (*p* <0.02), were positively associated with age, and negatively related to BMI (Figure 2). In the 63 NGT, normal-weight (i.e., BMI ≤ 25 kg/m^2^) subjects aged <65 years (42 women, 21 men), plasma NAA ranged 143–407 nmol/L (median 204, IQR 110 nmol/L) in men and 101–378 nmol/L (median 239, IQR 95) in women. By adjusting the entire data set for gender, age, and BMI, plasma NAA levels were inversely related to both FPG and HbA_1c_ (Figure 3); in this model, age was still a positive (*p* <0.0001), and BMI a negative (*p* = 0.044), covariate. To characterize the clinical phenotype of subjects with low plasma NAA levels, we split the NAA distribution into quartiles. In the lower quartile of plasma NAA levels there were more women, a younger age and higher BMI, and a worse glycemic control (Table 2).

**Table 1:** Characteristics of the study subjects^*^.

	**NGT**		**T2D/IGT**	
	**Men**	**Women**	**Men**	**Women**
*N*	60	96	186	163
Age (years)	50 ± 12	45 ± 12	58 ± 9	55 ± 10
BMI (kg/m^2^)	29.5 ± 8.8	37 ± 11.1	32.0 ± 5.3	33.5 ± 6.0
HbA_1c_ (%)	5.9 ± 1.7	5.7 ± 0.6	8.2 ± 1.4	8.6 ± 1.5
FPG (mg/dL)	93 ± 19	92 ± 17	158 ± 49	171 ± 61
NAA (nmol/L)	249 [113]	227 [88]	249 [67]	239 [75]

* *entries are mean ± SD or median [interquartile range]; BMI, body mass index; FPG, fasting plasma glucose; NAA, N-acetylaspartate*.

**Table 2:** Clinical phenotype of subjects by quartile of NAA distribution^*^.

	**Plasma NAA distribution**		
	**Bottom 25%**	**Upper 75%**	***p***
NAA (nmol/L)	129 [41]	260 [66]	<0.0001
Women (%)	61	48	0.0138
Age (years)	48 ± 11	56 ± 11	<0.0001
BMI (kg/m^2^)	34.2 ± 9.3	32.5 ± 6.6	0.07
HbA_1c_ (%)	8.2 ± 2.1	7.7 ± 1.6	0.0366
FPG (mg/dL)	158 ± 69	146 ± 53	0.09

* *entries are mean ± SD or median [interquartile range]; BMI, body mass index; FPG, fasting plasma glucose; NAA, N-acetylaspartate*.

In the 55 subjects (45 NGT and 10 T2D) who underwent bariatric surgery, the change in BMI at 6 months post-surgery averaged −11.5 ± 3.6 kg/m^2^ (*p* <0.0001), and was similar in NGT and T2D. Fasting plasma glucose dropped by 10 ± 16 mg/dL in NGT and by 95 ± 77 mg/dL in T2D (*p* <0.0001 for both), and plasma NAA increased by 18 (37) μmol/L (*p* <0.02).

Out of the 265 T2D patients, 192 individuals were randomized to receive dapagliflozin + placebo (Dapa+Plb), exenatide once weekly (EQW), or the combination (EQW+Dapa) in a 1:1:1 ratio for 1 year. At 52 weeks, NAA values were higher than at baseline [248 [94] vs. 239 [83] nmol/L, delta = 13 (38), *p* = 0.0013] across treatment. Each treatment resulted in significant reductions in BMI, fasting glucose, and HbA_1c_, and an increase in plasma NAA; the changes were (for BMI, FPG, and NAA) or tended to be (for HbA_1c_) accentuated with the combination as compared to either agent alone (Table 3). In the pooled data from all treatment arms, the changes in plasma NAA were inversely related to the concomitant changes in both HbA_1c_ (Figure 4). The regression predicts a mean NAA increase of 16 nmol/L for a 2% decrement in HbA_1c_.

**Table 3:** Variables by time and treatment^#^.

	**Baseline**	**1 year**	***p1***	***p2***	***p3***
Fasting glucose (mg/dL)			<0.0001	0.374	0.0214
Dapa + Plb (*n* = 70)	190 ± 42	145 ± 44			
EQW + Plb (*n* = 62)	190 ± 53	138 ± 37			
EQW + Dapa (*n* = 60)	194 ± 60	123 ± 32			
BMI (kg/m^2^)			* <0.0001*	0.412	0.0004
Dapa + Plb	33.1 ± 6.1	32.3 ± 5.9			
EQW + Plb	31.6 ± 5.4	31.5 ± 5.5			
EQW + Dapa	33.6 ± 6.4	32.1 ± 6.0			
HbA1c (%)			* <0.0001*	0.249	0.084
Dapa + Plb	9.3 ± 1.0	7.7 ± 1.1			
EQW + Plb	9.3 ± 1.0	7.6 ± 1.3			
EQW + Dapa	9.3 ± 1.1	7.2 ± 1.3			
NAA (nmol/L)			*0.0005*	0.539	0.035
Dapa + Plb	242 [97]	245 [100]			
EQW + Plb	236 [82]	248 [73]			
EQW + Dapa	238 [88]	248 [110]			

# *values are mean ± SD or median [IQR]; p^1^ = time, p^2^ = treatment, p^3^ = time x treatment by MANOVA for repeated measures*.

**Figure 4:**
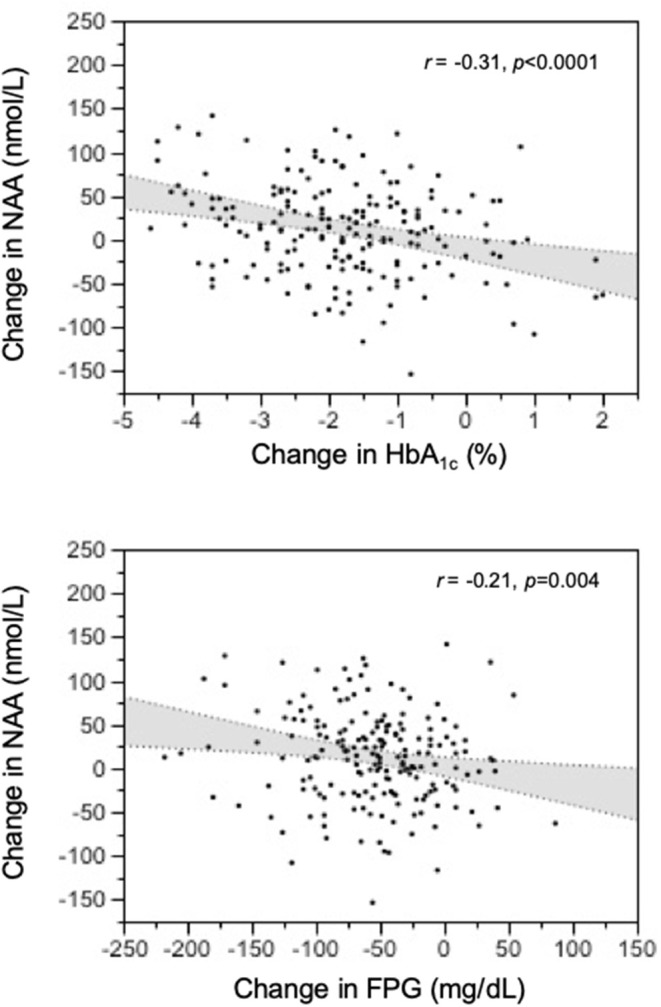
Relation of treatment-induced changes in plasma N-acetylaspartate (NAA) concentrations to the corresponding changes in HbA_1c_ and fasting glucose levels at 1-year post-treatment.

## Discussion

The data of this study allowed us to establish normative circulating NAA metrics, namely, median fasting concentrations of 230 nmol/L in non-obese, non-diabetic subjects below 65 years of age, and consistent, if not large, deviations with age and obesity (in opposite direction). Additionally, we report for the first time an inverse relation of plasma NAA to glycemic indices—HbA_1c_ and fasting glucose—even after controlling for sex, age, and BMI. The intervention protocols provided a prospective confirmation of this relationship by showing a consistent increase in plasma NAA concentrations following antidiabetic treatment (Figure 3).

It should be emphasized that no information is available on the acute changes in circulating NAA following physiological stimuli, e.g., a meal or a bout of physical exercise. More importantly, changes in plasma levels could reflect changes in NAA production/spillover from the brain into the bloodstream or changes in NAA removal from the plasma (or a combination thereof) (26). Thus, dysfunctional neurons might synthetize less NAA and/or the activity of the NAA-cleaving enzyme in glial cells [but also in brown adipocytes (39) and enterocytes (40)] might be increased: either process would reduce circulating NAA concentrations. The resulting clinical phenotype would resemble that profiled in subjects with lower NAA concentration (Table 3).

With regard to the effect of age, our finding of an independent, direct relationship between plasma NAA and age conflicts with two studies from the same group showing an age-related decline in serum NAA levels (41, 42). In the latter studies, however, the measured NAA concentrations were in the millimolar range, whereas reported values—including ours—are in the nanomolar range (34). Therefore, rather than a reflection of increased neuronal mass/function [i.e., an implausible correlate of aging (43)], our finding may suggest an age-related decrease in aspartoacylase, resulting in higher NAA levels. The extreme of this phenomenon would be Canavan disease, an autosomal-recessive mutation of the aspartoacylase gene associated with severe neurodegeneration (44).

The current finding of decreasing plasma NAA levels with increasing BMI is in line with the suggested role of obesity in NAA metabolism. By magnetic resonance spectroscopy (MRS), a higher BMI is associated with lower concentrations of NAA in brain frontal, parietal, and temporal white matter as well as in frontal gray matter and hippocampus (45, 46), and with progressive neuronal impairment and loss. Obesity can lead to astrogliosis, which is characterized by an increase in the number of primary astrocytic processes as well as in the length of these processes, leading to a more extensive contact between these cells and the local vasculature (47, 48). Astrocytes can be activated early after high-fat intake (49, 50) as a neuroprotective response to the rise in the concentration of fatty acids reaching the brain via the BBB (51–53). These structural changes could modify the ability of astrocytes to transport substances from the brain to the circulation, including a reduced release of NAA (54). Several studies have documented changes in urinary NAA excretion or expression of NAA producing enzymes in association with obesity and/or diabetes (39, 40, 55–57). In the present study, the body weight reduction induced by bariatric surgery was associated to a significant increase in circulating NAA. One can therefore speculate that weight loss might have had multiple favorable consequences on central and peripheral NAA metabolism, including the reduction of brain astrogliosis.

Hyperglycemia dramatically increases neuronal glucose levels, which leads to neuronal damage, a phenomenon known as glucose neurotoxicity (58). Several mechanisms have been proposed for glucose neurotoxicity, including glucose-driven oxidative stress, and protein glycation. In line with this *in vitro* evidence, it has been shown that NAA/creatine ratio is decreased in the frontal brain cortex in diabetic patients with poor glycemic control (25). High glucose has a dramatic impact on astrocyte phenotype by inhibiting its proliferation and disrupting its energy flow (59, 60). Moreover, high glucose is associated with an increased expression and secretion of inflammatory cytokines (interleukin-6 and interleukin-8) by astrocytes (61). These hyperglycemia-induced abnormalities of astrocyte-neuron coupling may underlie the reduced central NAA concentrations and NAA spill-over into the peripheral circulation.

Although peripheral NAA levels alone cannot be taken to reflect spill-over or astrocyte-neuron coupling, our finding of consensual changes in glycemic indices and plasma NAA (Figure 3) does lend support to the possibility that long-term changes in circulating NAA may be markers for brain dysfunction. Clearly, simultaneous measurements of plasma NAA, brain NAA signal in relevant areas, and cognitive function before and after antihyperglycemic treatment would conclusively establish origin and value of peripheral NAA assessment.

Of note is that glucagon-like peptide 1 (GLP-1) receptors are expressed in neurons, in particular pyramidal neurons in the hippocampus and neocortex, which suggests that they may play a role in neuronal activity and synaptic transmission (62–64). Glia cells also express the receptor (65). GLP-1 analogs have demonstrated an impressive range of protective effects on neurogenesis, synaptogenesis, cell repair, and reduced inflammation, which is consistent with the profile of growth factor activity (37, 66–70). In this context, it is intriguing that patients in either EQW treatment arm showed a significantly greater change in plasma NAA than patients in the Dapa+placebo arm (Table 2), suggesting a potential effect of GLP-1 receptor agonists/analogs independently of the glucose lowering effect (38, 71).

The strength of the study is based on a large population including subjects with a wide range of age and body weight, and well-characterized in terms of glucose metabolism and comorbidity. In both T2D and morbidly obese groups, psychiatric disorders, intellectual disability, severe cognitive impairment, neurodegenerative diseases, epilepsy, depression treatment, and traumatic brain injury over the preceding months were exclusion criteria, which reduced the chances of NAA alterations being due to specific brain abnormalities.

The main limitation of the present study is its retrospective nature. Consequently, the NAA associations we describe, whether cross-sectional or longitudinal, do not necessarily intimate a causative role of glucose metabolism for plasma NAA or a direct relation of plasma NAA to brain NAA metabolism. The results of the study provide preliminary, inferential evidence, which remains to be confirmed by multilevel prospective studies.

## Data Availability Statement

All datasets generated for this study are included in the article/supplementary material.

## Ethics Statement

The studies involving human participants were reviewed and approved by University of Pisa. The patients/participants provided their written informed consent to participate in this study. Written informed consent was obtained from the individual(s) for the publication of any potentially identifiable images or data included in this article.

## Author Contributions

EF and GD conceived the experimental design, analyzed the data, wrote and edited the manuscript. AS developed the tandem mass spectrometry-based assay and directed its validation. BC and SC performed LC-MS. AC and LG provided clinical samples. SD and RE provided feedback and contributed to the write-up and review of the manuscript. EF directed the study and is the guarantor of this work and, as such, had full access to all the data in the study and takes responsibility for the integrity of the data and accuracy of the data analysis. All authors read and approved the final manuscript.

## Conflict of Interest

SD has served on advisory panels for Abbott, AstraZeneca, Boehringer Ingelheim, Eli Lilly and Co., GlaxoSmithKline, Merck & Co., Novartis Pharmaceuticals, Novo Nordisk, Sanofi, Laboratoires Servier, and Takeda Pharmaceuticals and been given research support from AstraZeneca, Boehringer Ingelheim, Merck & Co., and Novartis Pharmaceuticals. RE is an employee of and holds stock in AstraZeneca. EF has participated in scientific advisory boards for Boehringer Ingelheim, Eli Lilly, and Sanofi; has performed *ad hoc* consulting for Janssen, AstraZeneca, Mitsubishi Tanabe; has participated in occasional speaking engagements for AstraZeneca, Novo Nordisk, Sanofi, Mitsubishi Tanabe, Eli Lilly, Boehringer Ingelheim, Merck Sharp & Dohme; and has received research grant support from Boehringer Ingelheim and AstraZeneca. The remaining authors declare that the research was conducted in the absence of any commercial or financial relationships that could be construed as a potential conflict of interest.

## Abbreviations

NAA: N-acetylaspartate
T2DM: type 2 diabetes
IGT: impaired glucose tolerance
NGT: normal glucose tolerance
RYGB: Roux-en-Y Gastric Bypass
FPG: Fasting plasma glucose
MRS: magnetic resonance spectroscopy
BBB: blood-brain barrier
GLP-1RAs: GLP-1 receptor agonists/analogs
Dapa+Plb: dapagliflozin + placebo
EQW: exenatide once weekly
EQW+Dapa: exenatide-dapagliflozin combination.
